# Coronary allograft vasculopathy managed by Flash ostial balloon in a pediatric patient

**DOI:** 10.21542/gcsp.2021.27

**Published:** 2021-12-31

**Authors:** Viral K. Desai, Mohammad Mathbout, Apurv Agarwal, Ibrahim Fahsah, Shahab Ghafaghazi

**Affiliations:** 1 Department of Internal Medicine, University of Louisville, Louisville KY; 2 Department of Cardiovascular Medicine, University of Louisville, Louisville KY

## Abstract

Coronary allograft vasculopathy (CAV) is the most significant cause of morbidity and mortality in heart transplant recipients. Inflammation and endothelial dysfunction caused by graft rejection and viral infections leads to a combination of circumferential intimal fibromuscular hyperplasia, atherosclerosis, and inflammation affecting all layers of the vessel wall. Though obstructive CAV is often asymptomatic, posing a diagnostic challenge in post-transplant patients, early diagnosis and treatment aid faster recovery and improved outcomes. The role of percutaneous coronary intervention in the treatment of CAV is unknown and not well studied in the pediatric population. We present a first-in-human case of ostial left main coronary artery disease managed with flaring of the ostial coronary stent using a Flash ostial balloon in a pediatric patient with history of an orthotopic heart transplant.

## Introduction

Coronary allograft vasculopathy (CAV) is an accelerated form of arteriopathy affecting the coronary vessels of both pediatric and adult orthotopic heart transplant (OHT) recipients. It is the most significant cause of morbidity and mortality following a heart transplant^1^. Though immunosuppressive therapy is the mainstay of treatment for CAV, the role of percutaneous coronary intervention (PCI) remains unknown and data, especially in the pediatric population, appears scarce. We present the case of an 8-year-old female presenting with cardiogenic shock, two years after an OHT, who was found to have severe left main coronary artery disease thought to be due to CAV. To our knowledge, this is the first-ever case of CAV described in the literature which was managed with flaring of the ostial coronary stent using a Flash ostial balloon.

## Case

An 8-year-old female with a history of a double inlet left ventricle, dextro-transposition of great arteries status post-OHT, presented with fever and fatigue. On physical examination, she appeared somnolent, had a temperature of 101.4°F, and was hypotensive with a blood pressure of 62/44 mmHg. On admission, she was intubated and was started on broad-spectrum antibiotics and empiric antiviral therapy as she was presumed to be in septic shock. She required vasopressors and ionotropic support with milrinone, however, she deteriorated further and was cannulated for extracorporeal membrane oxygenation (ECMO).

Even though her previous surveillance cardiac biopsy was unremarkable, given her history of acute graft rejection and poor graft function on transesophageal echocardiography with an ejection fraction of 15%, intravenous steroids and plasmapheresis were initiated.

On day 7, her blood cultures remained negative, although she tested positive for coronavirus NL63. She was also found to have DNA detectable for CMV and parvovirus B19 but not at levels to cause an infection. She improved clinically after receiving 7 days of empiric antimicrobial treatment and IV steroids. On day 8, she was extubated and decannulated from ECMO.

**Figure 1.**
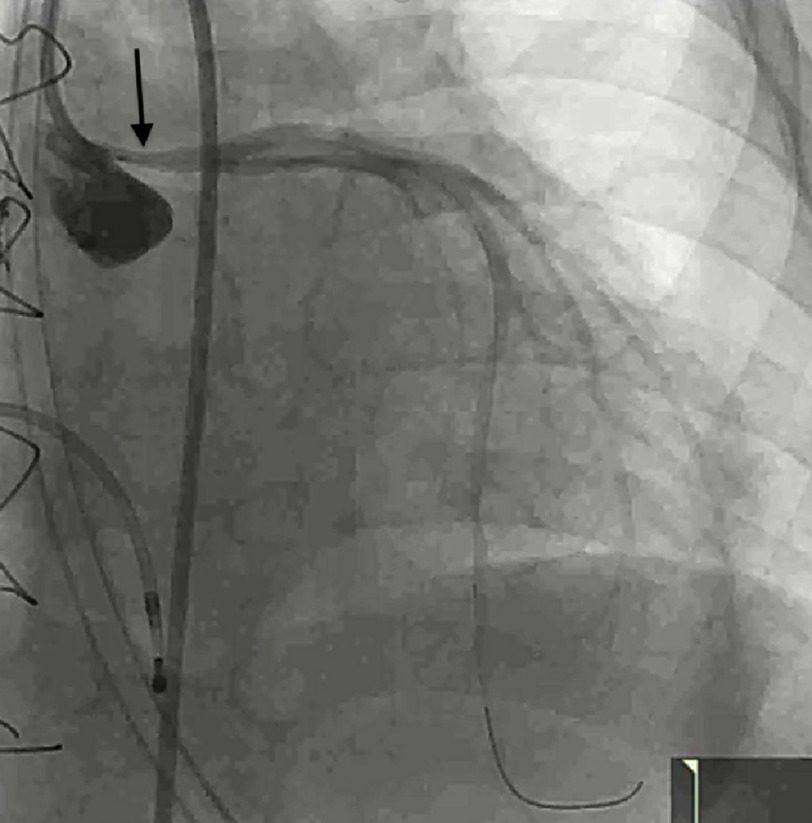
Coronary angiography (LAO 17*/CRAN 24*) showing severely stenotic ostial left main artery.

On day 15, owing to the previous history of graft rejection, a presentation of cardiogenic shock meant she was taken to the catheterization lab for coronary angiography and right heart catheterization, as well as a right ventricular (RV) endomyocardial biopsy. The coronary angiography revealed a hemodynamically significant ostial left main (LM) coronary artery stenosis [Figure 1]. The RV endomyocardial biopsy showed mild ISHLT grade 1R acute cellular rejection but was negative for donor-specific antibody and antibody-mediated rejection. After discussion with the heart team, it was decided to go ahead with a diagnostic intravascular ultrasound (IVUS) followed by a percutaneous coronary intervention, using a Flash ostial system (Ostial Corporation, Sunnyvale, CA).

**Figure 2.**
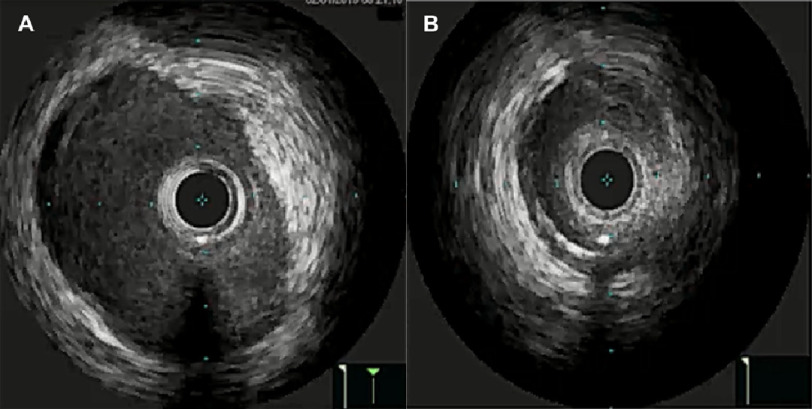
(A) - Intra-vascular ultrasound (IVUS) images obtained following intra-coronary infusion of 200mcg of nitroglycerin. Images demonstrate the distal reference area of the left main, measured at 16 mm^2^; (B) - In comparison to minimal luminal area (MLA), measured at 5.6 mm^2^.

IVUS images were obtained in the cardiac catheterization lab, which demonstrated the distal reference area of the left main artery as 16 mm^2^ [Figure 2A] in comparison to a minimal luminal area measuring 5.6 mm^2^ [Figure 2B].

**Figure 3.**
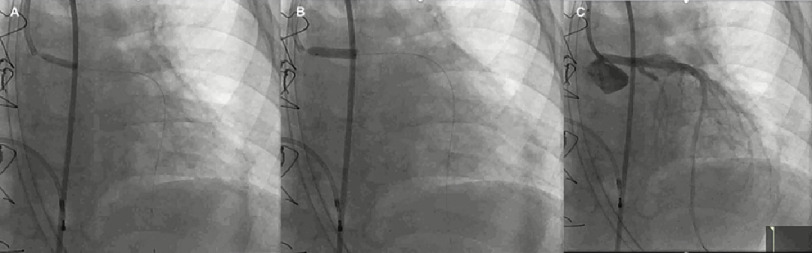
Images demonstrate various stages of the intervention. (A) - pre-dilating with 3.0 × 15 NC balloon; (B) Deployment of Xience 3.5 × 15 mm stent; (C) Angiography post drug eluding stent placement (LAO 18*/CRAN 25*).

**Figure 4.**
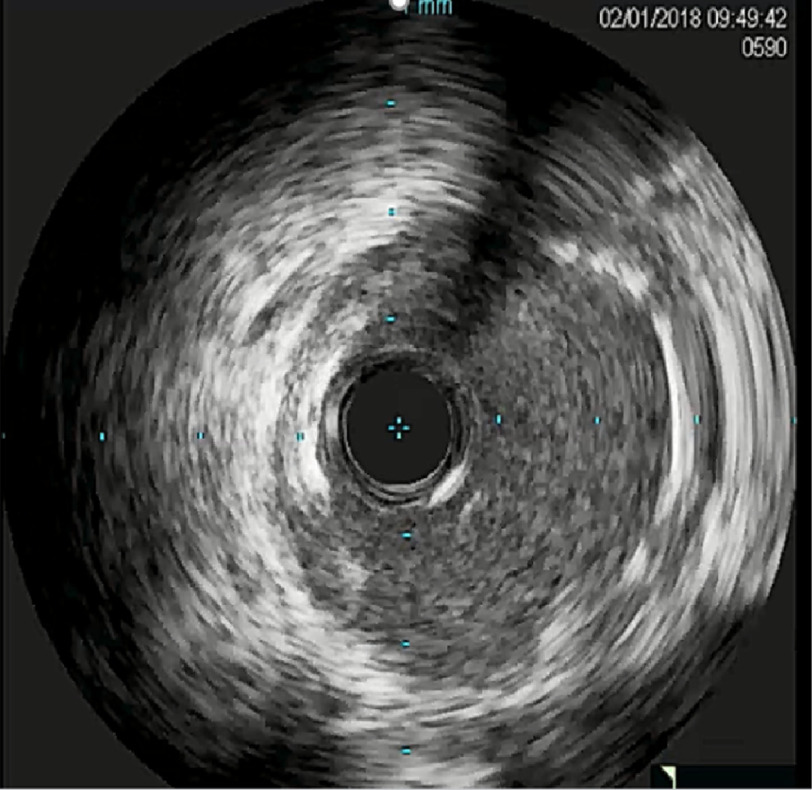
Intra-vascular ultrasound (IVUS) images obtained following stent placement, demonstrating incomplete stent apposition (ISA).

**Figure 5.**
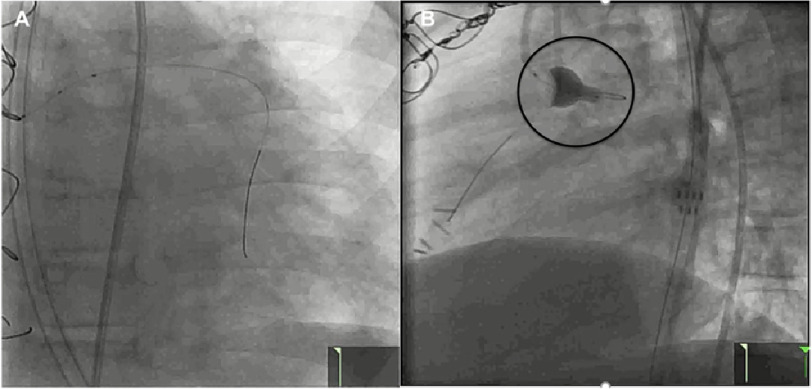
(A) - Images demonstrate stent post-dilation with 4.0 then 4.5 mm balloon - showing wire markers; (B) - LAO 19*/CRAN 11*), followed by ostial Flash balloon up (LAO 86*/CRAN 5*), to ensure adequate stent apposition.

The Flash ostial system was used for intervention with deployment of Xience 3.5 × 15 mm stent [Figure 3]. Since repeat IVUS showed incomplete stent apposition [Figure 4], 4 mm then 4.5 mm balloons were used for dilation followed by ostial Flash balloon to ensure adequate stent apposition [Figure 5].

The patient improved rapidly, and the graft function improved on echocardiogram. She was discharged on dual antiplatelet therapy, with aspirin and clopidogrel. Following her discharge, the patient did well and has since followed up as an outpatient.

## Discussion

Cardiac allograft vasculopathy remains the most significant cause of morbidity and mortality after OHT^1^. According to the data from the registry of the International Society for Heart and Lung Transplantation, CAV is the foremost cause of mortality between 1 and 3 years of transplant and develops in about one-third of recipients of OHT within 10 years^2^.

Compared to adults the incidence of CAV is lower in children, with patients aged >12 years having a 2.9-fold higher risk for developing CAV compared to children transplanted at <5 years of age^2^. This lower incidence in the pediatric population may be related to a lack of donor/recipient atherosclerotic risk factors or preexisting coronary endothelial injury^2^.

Although the exact underlying pathogenesis remains unclear, it is likely secondary to a combination of immune and non-immune mediated phenomena leading to a combination of circumferential intimal fibromuscular hyperplasia, atherosclerosis, and inflammation affecting all layers of the vessel wall^2,3^. Antibodies against mismatched donor antigens, which are formed in some patients, play a major role in the development of immunologic injury of the coronary arteries. These donor-specific antibodies constitute an independent risk factor for the development of CAV and graft rejection^4^.

Obstructive CAV is often asymptomatic, due to the absence of typical angina symptoms resulting from the denervation of the donor’s heart during transplant, thus, posing a diagnostic challenge in post-transplant patients. Various invasive and non-invasive modalities are used in cases with suspected CAV. Coronary angiography is traditionally used as the primary investigation for suspected CAV, but it is limited to examining the vessel lumen and not the vessel wall^5^. Intra-vascular ultrasound, on the other hand, allows accurate estimation of vessel wall thickening along with focal narrowing, thus having a higher sensitivity for early detection of CAV ^6^.

In a cohort of 31 adult patients with OHT, Torres et al. demonstrated CAV in 54.8% of patients via IVUS, compared to 32% via coronary angiography^7^. Various other studies have also established the superiority of IVUS over angiography for early detection of CAV^8^. Optical coherence tomography (OCT) is another modality that can detect early intimal hyperplasia even below the limit of IVUS (150 μm) ^9^.

Early diagnosis of CAV is essential for appropriate and timely management and to prevent the progression of the disease. Once diagnosed, the options are limited to immunosuppressive therapy, percutaneous coronary intervention (PCI)/revascularization, or re-transplantation.

Immunosuppressive therapy is the mainstay for the treatment and prevention of CAV. As per the International Society of Heart and Lung Transplantation Guidelines, the standard post-transplant regimen includes calcineurin inhibitors, along with mycophenolate mofetil or azathioprine. Everolimus and sirolimus have been shown to reduce the incidence and delay the progression of CAV and can replace azathioprine/mycophenolate mofetil in suspected cases.

Routine use of statins, regardless of hyperlipidemia, is advocated for all ages after OHT to prevent CAV along with risk factor modification^3^. The role of percutaneous coronary intervention in the treatment of CAV is unknown. Although a recent study by Ullah et al. showed that PCI in CAV may be associated with increased in-hospital complications^10^, this was studied in adults, and data on pediatric population is scarce. Nonetheless, PCI can also be performed in selected focal coronary lesions limited to a single coronary artery, as a palliative measure^11^.

In case of technically challenging ostial lesions, one option for PCI is the use of the Flash Ostial Balloon System. This dual balloon design combines a higher-pressure dilatation balloon with an oversized low-pressure anchoring balloon. Due to the similarity with the natural anatomy of the funnel-shaped ostium, the Flash ostial system is used to maintain the position of the catheter at the aorto-ostial junction and to achieve excellent stent wall apposition post-dilatation in technically challenging ostial lesions. The Flash ostial system has previously been used for ostial lesions occurring after coronary artery bypass grafting ^12^ and aorto-ostial lesions with minimal complications^13^.

This patient presented with cardiogenic shock and was found to have mild rejection on endomyocardial biopsy and CAV on CCTA and coronary angiography, verified on IVUS. It is interesting to note that even though the risk of developing CAV increases in pediatric patients who have experienced ≥2 rejection episodes during the first post-transplant year, or a rejection episode >1-year post-transplant, clinical rejection appears to pose a much higher risk of developing CAV compared to rejection detected on routine surveillance biopsy. Thus, even though our patient only showed mild rejection on endomyocardial biopsy, her presentation could have been secondary to clinical graft rejection which was treated with plasmapheresis and steroids.

The ostial lesion was stented using the Flash ostial system which has not been previously described in the literature. The ostial Flash balloon system was chosen to achieve optimal ostial stent apposition, permitting adequate revascularization. Furthermore, realizing that repeat ischemic evaluation is likely needed in the future, use of the ostial Flash system will allow smoother catheter engagement and angiography, by “crushing” the protruding stent struts against the aorta. Management of in-stent restenosis, and under sizing with subsequent vessel growth, will be technical and highly dependent on the anatomical aspects of the patient’s presentation. Coronary imaging (IVUS/OTC) would likely be of good use.

### What we have learned?

An incomplete understanding of pathogenesis of CAV has limited the development of therapies directed at process reversal or prevention. Clinical transplant rejection can cause a higher risk of developing CAV compared to rejection just seen on protocol biopsy. In case of technically difficult coronary artery lesions caused by CAV novel approaches like flash ostial system can be used.

## Abbreviations

CORV: Coronary Allograft Vasculopathy
PCIC: Percutaneous Coronary Intervention, Complex PCI
IVUS: Imaging, Intravascular Ultrasound
TRAN: Transplantation
